# Relação entre o Realce Tardio pelo Gadolínio e os Parâmetros de Repolarização Ventricular em Pacientes com Insuficiência Cardíaca com Fração de Ejeção Reduzida

**DOI:** 10.36660/abc.20200149

**Published:** 2021-06-25

**Authors:** Ali Riza Demir, Omer Celik, Songül Ustündağ, Begum Uygur, Mustafa Umut Somuncu, Emre Yilmaz, Yalcin Avci, Gokhan Demirci, Serkan Kahraman, Mehmet Erturk

**Affiliations:** 1 Departamento de Cardiologia University of Health Science Istanbul Mehmet Akif Ersoy Thoracic and Cardiovascular Surgery Training and Research Hospital Istambul Turquia Departamento de Cardiologia , University of Health Science , Istanbul Mehmet Akif Ersoy Thoracic and Cardiovascular Surgery Training and Research Hospital , Istambul - Turquia; 2 Departamento de Cardiologia Erzincan Binali Yıldırım University Mengücek Gazi Training Research Hospital Erzincan Turquia Departamento de Cardiologia , Erzincan Binali Yıldırım University Mengücek Gazi Training and Research Hospital , Erzincan - Turquia; 3 Departamento de Cardiologia Zonguldak Bülent Ecevit University Faculty of Medicine Zonguldak Turquia Departamento de Cardiologia , Zonguldak Bülent Ecevit University Faculty of Medicine , Zonguldak - Turquia; 4 Departamento de Cardiologia Görele Op. Dr. Ergun Ozdemir State Hospital Giresun Turquia Departamento de Cardiologia , Görele Op. Dr. Ergun Ozdemir State Hospital , Giresun - Turquia

**Keywords:** Insuficiência Cardíaca, Gadolínio, Volume Sistólico, Diagnóstico por Imagem, Espectroscopia de Ressonância Magnética/métodos, Eletrocardiografia/métodos, Acompanhamento dos Cuidados da Saúde, Ética

## Abstract

**Fundamento:**

A insuficiência cardíaca com fração de ejeção reduzida (ICFEr) é uma doença de alta prevalência que requer hospitalizações repetidas e causa morbimortalidade significativa. Portanto, o reconhecimento precoce de preditores de resultados desfavoráveis é essencial para o manejo do paciente.

**Objetivo:**

O objetivo do presente estudo é investigar a relação entre realce tardio pelo gadolínio (RTG) detectado por ressonância magnética cardíaca (RMC) e os parâmetros de repolarização, como o intervalo QT corrigido (QTc), intervalo Tp-e, ângulo QRS-T frontal detectado pelo eletrocardiograma (ECG) de 12 derivações na ICFEr.

**Método:**

Neste estudo observacional, retrospectivo, de centro único, foram incluídos 97 pacientes consecutivos com ICFEr submetidos à RMC. A população do estudo foi dividida em dois grupos, de acordo com a presença de RTG. Foram registradas medidas ecocardiográficas e de RMC e características demográficas. Os intervalos QTc, intervalos Tp-e, e ângulos QRS-T frontais foram calculados a partir do ECG. Um valor de p <0,05 foi considerado estatisticamente significativo.

**Resultados:**

O RTG foi detectado em 52 (53,6%) de 97 pacientes com ICFEr. Os intervalos QTc (p=0,001), intervalos Tp-e (p<0,001), e os ângulos QRS-T frontais (p<0,001) foram significativamente maiores no grupo RTG quando comparados ao grupo não-RTG. Na análise de regressão univariada realizada para investigar os preditores de RTG na ICFEr, todos os três parâmetros de repolarização alcançaram valores significativos, mas na análise multivariada o único parâmetro de repolarização que permaneceu significativo foi o intervalo Tp-e (OR = 1,085 IC 95% 1,032-1,140, p=0,001).

**Conclusão:**

Com o prolongamento do intervalo Tp-e, pode-se prever a presença de fibrose miocárdica, a qual é um substrato arritmogênico, em pacientes com ICFEr.

## Introdução

A insuficiência cardíaca (IC) é uma doença de alta prevalência na população em geral, que requer repetidas hospitalizações e causa significativa morbidade e mortalidade. ^1 , 2^ Como consequência desses graves desfechos, a IC resulta em alto ônus econômico para os sistemas de saúde. Portanto, o reconhecimento precoce de preditores de resultados desfavoráveis é essencial para o manejo do paciente. A mortalidade ocorre comumente devido à falha do bombeamento cardíaco ou episódios arritmogênicos. ^3 , 4^ Seja de origem isquêmica ou não isquêmica, os pacientes com IC apresentam mais fibrose miocárdica do que os indivíduos saudáveis, a qual é substrato para arritmias e remodelamento negativo, o que diminui gradativamente a função ventricular esquerda. ^5 - 7^

O realce tardio pelo gadolínio (RTG) por ressonância magnética cardíaca (RMC) é capaz de detectar anormalidades teciduais, particularmente fibrose miocárdica. ^8^ Pesquisadores descobriram recentemente que, além da FEVE, o RTG pode ser utilizado como um marcador de prognóstico desfavorável em pacientes com IC. ^9 - 11^

Na prática clínica, o ECG é amplamente utilizado para prever o risco arritmogênico. O intervalo QTc, o intervalo Tp-e e o ângulo QRS-T são parâmetros de repolarização ventricular, recomendados como preditores de arritmias ventriculares malignas. O Tp-e é um parâmetro relativamente novo para a dispersão transmural da repolarização e também está relacionado ao risco de morte súbita cardíaca (MSC). ^12 , 13^ O ângulo QRS-T frontal, definido como um ângulo entre as direções de despolarização e repolarização ventricular, reflete a heterogeneidade elétrica e alterações anormais na direção da sequência de repolarização, devido a anormalidades estruturais cardíacas, e o ângulo QRS-T frontal é um forte preditor de instabilidade elétrica e MSC. ^14 - 16^

Nosso objetivo foi investigar a relação entre o RTG detectado por RMC e os parâmetros de repolarização, como o intervalo QTc, o intervalo Tp-e e o ângulo QRS-T frontal, detectados por ECG de 12 derivações em pacientes com ICFEr, e determinar qual desses parâmetros é mais significativo neste aspecto.

## Métodos

### População do estudo

Este estudo incluiu 97 pacientes com ICFEr admitidos na clínica de cardiologia entre janeiro de 2017 e junho de 2019, submetidos a RMC por vários motivos (pesquisa da etiologia da IC, pesquisa de viabilidade, cálculo de FE etc.) e que apresentavam FE <40% no ecocardiograma transtorácico.

Foram registrados dados sobre as características demográficas dos pacientes (idade, sexo e índice de massa corporal), histórico médico [diabetes mellitus (DM), hipertensão (HT), hiperlipidemia (HL) e doença arterial coronariana], medicamentos [betabloqueadores, inibidores da enzima conversora de angiotensina (IECA), bloqueadores do receptor de angiotensina (BRA), espironolactona, diuréticos, digoxina, estatinas], ritmo cardíaco [ritmo sinusal, fibrilação atrial e bloqueio de ramo esquerdo (BRE)], resultados de exames ecocardiográficos, ECG de 12 derivações e exames bioquímicos no sangue. A classe funcional da *New York Heart Association* (NYHA) de cada paciente foi determinada.

Pacientes com idade <18 anos ou >90 anos; com histórico de síndrome coronariana aguda ou intervenção coronária primária nos últimos 6 meses; e que apresentavam hipotensão, edema pulmonar ou choque cardiogênico, foram excluídos do estudo. Além disso, também foram excluídos pacientes com doença renal crônica (DRC) em estágio 4-5; aqueles com foco ativo de infecção, doença neurológica grave o suficiente para afetar os resultados bioquímicos e hematológicos, doença pulmonar obstrutiva crônica (DPOC), malignidade ou comprometimento da função hepática/insuficiência hepática.

O estudo foi aprovado pelo comitê de ética do Mehmet Akif Ersoy Thoracic and Cardiovascular Surgery Training Research Hospital. Este estudo foi realizado de acordo com os requisitos da Declaração de Helsinque.

### Eletrocardiograma

O ECG de 12 derivações foi realizado com velocidade do papel de 25 mm/s com o paciente em repouso em posição supina. A freqüência cardíaca de repouso foi então medida a partir dos dados do ECG. Todos os dados do ECG foram digitalizados e transferidos para um computador pessoal e utilizados para ampliação de 400% com o software Adobe Photoshop (Adobe Systems, Inc., San Jose, CA, EUA). As medidas do ECG dos intervalos QTc, Tp-e e ângulo QRS-T frontal foram realizadas por 2 cardiologistas que desconheciam os dados da RMC dos pacientes. Os sujeitos com onda U no ECG foram excluídos do estudo. O valor médio de 3 exames foi calculado para cada derivação. O intervalo QT foi medido do início do complexo QRS ao final da onda T. Em pacientes com BRE, foi utilizada ^17^ a fórmula [QT = QTBRE - (0,86 x QRSBRE - 71)] recomendada por Wang et al., e o intervalo QT foi corrigido pela frequência cardíaca usanso a fórmula de Bazett [QTc = QT√ (intervalo R – R)] . O intervalo Tp-e foi definido como o intervalo do pico da onda T até o final da mesma. As medidas do intervalo Tp-e foram realizadas a partir das derivações precordiais. A relação Tp-e/QT e a relação Tp-e/QTc foram calculadas a partir dessas medidas. O ângulo QRS-T frontal foi calculado como o valor absoluto da diferença entre os eixos do do complexo QRS e das ondas T no plano frontal. Se tal diferença fosse maior que 180°, o ângulo QRS-T era ajustado para o ângulo mínimo de 360° menos o valor absoluto da diferença entre os eixos do complexo QRS e das ondas T no plano frontal. ^18^ As diferenças intra e inter-pesquisadores para os intervalos QTc, Tp-e e ângulo QRS-T foram menores que 5%.

### Aquisição de imagem

As imagens foram adquiridas em scanners de 1,5 T (MAGNETON Aera, Siemens, Erlangen, Alemanha) com cobertura total do miocárdio. Sequências balanceadas de precessão livre no estado estacionário foram utilizadas para obter imagens em apneia em três planos de eixo longo, seguidas por uma série contígua de cortes no eixo curto do anel atrioventricular até o ápice. ^19^ Imagens de realce tardio foram adquiridas 10 min após a administração de 0,1 mmol/kg de agente de contraste de gadolínio intravenoso (Gadopentetato dimeglumina/gadobutrol, Bayer, Berlim, Alemanha) com uma sequência gradiente-eco preparada para inversão-recuperação. ^20^ Os tempos de inversão foram otimizados para ‘anular’ o miocárdio normal com imagens adquiridas em duas direções de codificação de fase ortogonal para excluir a presença de artefatos.

### Análise de imagem

As imagens foram transferidas para uma estação de trabalho (Leonardo, Siemens Medical Solutions, Erlangen, Alemanha) para análise. Para a análise funcional, foi utilizado o programa de software disponível comercialmente Argus (Siemens Healthcare, Erlangen, Alemanha).

### Análise de dados da RMC

As bordas endocárdicas e epicárdicas foram traçadas manualmente utilizando os dois sistemas de software, e a análise funcional foi realizada.

Na análise com o software Argus (Siemens Healthcare, Erlangen, Alemanha) para cada estudo, foram determinadas as fases diastólica e sistólica finais. Para a detecção de cada fase, foram utilizados os diâmetros maior e mais estreito da cavidade ventricular no meio do ventrículo. As bordas endocárdica e epicárdica foram traçadas manualmente em imagens no eixo curto em ambas as fases. As bordas do endocárdio foram traçadas utilizando-se a diferença de intensidade entre a câmara quando cheia de sangue e a intensidade moderada do miocárdio. Os músculos papilares foram incluídos na análise volumétrica do VE. Enquanto a borda epicárdica estava sendo detectada, o septo interventricular era incluído no volume do VE. O corte mais basal circundada por pelo menos 50% do miocárdio preenchido com sangue foi definida como o segmento basal do ventrículo esquerdo. Isso foi incluído no volume da câmara do VE. O ápice foi definido como o último corte com lúmen visível ao longo de todo o ciclo cardíaco. O volume sistólico final (VFS), volume diastólico final (VDF) e a FE foram determinados de acordo com a regra de Simpson. O tempo decorrido entre a entrada dos dados e a obtenção dos resultados foi calculado para cada paciente.

Imagens de RMC foram reanalisadas e documentadas utilizando o modelo cardíaco de 17 segmentos recomendado pela *American Heart Association* para melhorar a padronização dos resultados. O ventrículo esquerdo foi avaliado a partir das imagens do eixo curto dos segmentos basal, médio e apical. As cavidades basal e média foram divididas em 6 segmentos iguais: anterior, anterosseptal, ínfero-septal, inferior, ínfero-lateral e anterolateral. O segmento apical foi dividido em 4 segmentos: anterior, septal, inferior e lateral. O ponto mais apical foi denominado ápice e constituiu o 17º segmento. As imagens com contraste foram analisadas visualmente por dois observadores experientes que desconheciam outros dados da RM, dados ecocardiográficos e clínicos. O realce tardio pelo gadolínio foi classificado através de avaliação visual, e cada segmento foi classificado em uma escala de 2 pontos (escore de fibrose segmentar; 0 = ausência de realce tardio do gadolínio, 1 = presença de realce tardio pelo gadolínio), utilizando o método de Kaandorp et al. devido à frequência de realce linear e irregular em pacientes com cardiomiopatia dilatada não-isquêmica.

## Análise estatística

Os dados foram analisados utilizando o *Statistical Package for the Social Sciences* , versão 24.0 (SPSS Inc., Chicago, Illinois, EUA). No caso das variáveis com distribuição normal, a análise foi feita através de métodos visuais (histogramas, curvas de probabilidade) e analíticos (Kolmogorov-Smirnov). As variáveis contínuas com distribuição normal foram expressas como média ± desvio padrão (DP), as variáveis contínuas sem distribuição normal foram expressas como mediana (intervalo interquartil) e as variáveis categóricas como porcentagem (%). Variáveis contínuas como intervalo QTc, intervalo Tp-e e ângulo QRS-T foram avaliadas utilizando-se o teste *t* de Student não pareado e o teste U de Mann-Whitney entre os dois grupos. O teste do qui-quadrado ou o teste exato de Fisher foram usados para comparar variáveis categóricas. A correlação entre o intervalo QTc, intervalo Tp-e e ângulo QRS-T e as demais variáveis contínuas foram identificadas pelos testes de Pearson ou Spearman. A análise de regressão logística foi realizada para determinar os preditores independentes da presença de RTG na RMC em pacientes com ICFEr. Primeiramente foi realizada a análise de regressão logística univariada, e os parâmetros que foram significativos nesta análise (p<0,05) foram incluídos na análise de regressão logística multivariada. A análise da curva característica de operação do receptor (ROC) foi realizada para determinar os valores de corte. A predição significativa foi aceita quando a área sob a curva ROC foi maior que 0,5; um valor de p<0,05 foi aceito como estatisticamente significativo.

## Resultados

A média de idade dos 97 pacientes com ICFEr foi de 54,8 ± 13,8 anos. Nossa população do estudo incluiu 75 (77,3%) pacientes do sexo masculino e 22 (22,7%) do sexo feminino. Dividimos nossos pacientes em dois grupos de acordo com a presença de RTG na RMC. O RTG foi detectado em 52 (53,6%) pacientes. Os dados demográficos basais e os resultados laboratoriais para ambos os grupos são apresentados na Tabela 1 .

**Tabela 1:** – Características demográficas, clínicas e laboratoriais basais dos grupos de estudo

Variável	ICFEr sem RTG (n=45)	ICFEr com RTG (n=52)	Valor de p
Idade, anos	50,2±15,6	58,7±10,7	0,002
Sexo masculino, n (%)	26 (57,8%)	49 (94,2%)	<0,001
Índice de massa corporal, kg/m ^2^	27,7±4,2	26,6±3,7	0,171
Diabetes mellitus, n (%)	11 (24,4%)	17 (32,7%)	0,371
Hipertensão, n (%)	40 (88,9%)	46 (88,5%)	0,947
Doença arterial coronariana, n (%)	20 (44,4%)	45 (86,5%)	<0,001
Hiperlipidemia, n (%)	20 (44,4%)	36 (69,2%)	0,014
Frequência cardíaca, bpm	78,2±18,9	76,8±16,1	0,691
Fibrilação atrial, n (%)	5 (11,1%)	3 (5,8%)	0,466
BRE, n (%)	8 (17,8)	8 (15,4)	0,751
NYHA classe I, n (%)	10 (22,2%)	9 (17,3%)	0,543
NYHA classe II, n (%)	32 (71,1%)	36 (69,2%)	0,840
NYHA classe III, n (%)	3 (6,7%)	7 (13,5%)	0,331
Creatinina, mg/dL	0,80 (0,70-0,90)	1,10 (0,80-1,40)	<0,001
Taxa de filtração glomerular, ml/dk	91,3±25,1	71,9±26,3	0,001
Betabloqueadores, n (%)	42 (93,3%)	50 (96,2%)	0,661
IECA ou BRA, n (%)	40 (88,9%)	42 (80,8%)	0,270
Estatinas, n (%)	16 (35,6%)	36 (69,2%)	0,001
Diuréticos, n (%)	25 (55,6%)	31 (59,6%)	0,686
Espironolactona, n (%)	31 (68,9%)	29 (55,8%)	0,185
Digoxina, n (%)	5 (11,1%)	2 (3,8%)	0,244
Diltiazem, n (%)	3 (6,7%)	2 (3,8%)	0,661
Verapamil, n (%)	0 (0,0%)	1 (1,9%)	1,0

*Os dados são apresentados como porcentagem, média ± desvio padrão ou mediana (intervalo interquartil). ICFEr: insuficiência cardíaca com fração de ejeção reduzida, RTG: realce tardio do gadolínio, BRE: bloqueio do ramo esquerdo, NYHA: New York Heart Association, IECA: inibidor da enzima de conversão da angiotensina, BRA: bloqueador do receptor da angiotensina.*

A comparação dos parâmetros calculados a partir do ECG e das variáveis detectadas pela RMC são mostradas na Tabela 2 . O reflexo da repolarização no ECG, como os intervalos QT, QTc, intervalo Tp-e, relação Tp-e/QTc e ângulo QRS-T foram significativamente maiores no grupo com RTG. No grupo RTG, foi detectada uma média de 6,48 ± 3,54 segmentos que apresentaram realce.

**Tabela 2:** – Comparação dos grupos de estudo em relação a alguns parâmetros calculados a partir da RMC e ECG

Variável	ICFEr sem RTG (n=45)	ICFEr com RTG (n=52)	Valor de p
Duração de QRS, ms	103,6±26,2	101,4±20,9	0,650
Intervalo QT, ms	391,6±53,3	418,9±50,3	0,011
Intervalo QTc, Bazett	438,2±42,3	467,3±42,9	0,001
Intervalo Tp-e, ms	91,4±12,3	108,3±14,6	<0,001
Relação Tp–e/QT	0,235±0,034	0,261±0,042	0,001
Relação Tp–e/QTc	0,208±0,025	0,233±0,038	<0,001
Ângulo QRS-T, graus	61 (28-112)	136 (82-159)	<0,001
FEVE,%	30,9±10,7	28,8±8,3	0,275
DDFVE, mm	57,3±8,8	57,7±7,7	0,826
DSVE, mm	45,9±10,5	45,6±8,7	0,891
Índice VDFVE, mL/m ^2^	110,1±40,8	113,9±36,7	0,641
Índice VSFVE, mL/m ^2^	73,8±40,1	77,1±31,1	0,661
Número de segmentos VE		6,48±3,54	

*Os dados são apresentados como porcentagem, média ± desvio padrão ou mediana (intervalo interquartil). ICFEr: insuficiência cardíaca com fração de ejeção reduzida; RTG: realce tardio do gadolínio; FEVE: fração de ejeção do ventrículo esquerdo; DDVE: diâmetro diastólico final do ventrículo esquerdo; DSVE: diâmetro sistólico final do ventrículo esquerdo; VDFVE: volume diastólico final do ventrículo esquerdo; VSFVE: volume sistólico final do ventrículo esquerdo; VE: ventrículo esquerdo.*

A análise de correlação do intervalo QTc, intervalo Tp-e e ângulo QRS-T com outras variáveis foi mostrada na Tabela 3 . Houve correlações fracas entre o intervalo QTc e FEVE, DDFVE e DSFVE e também houve correlações fracas entre o ângulo QRS-T e FEVE, DDVE, DSVE, índice VDFVE e índice VSFVE. Embora não haja correlação entre o Tp-e e parâmetros da RMC, que mostram as estruturas e funções cardíacas, o melhor coeficiente de correlação entre o número de segmentos RTG foi obtido com o intervalo Tp-e. Houve uma correlação média com o intervalo Tp-e (r = 0,564, p <0,001) e correlações fracas com o intervalo QTc (r = 0,262, p = 0,009) e o ângulo QRS-T (r = 0,369, p<0,001). Outro achado de nosso estudo foi a existência de vários graus de correlações entre os próprios intervalo QTc, intervalo Tp-e e ângulo QRS-T.

**Tabela 3:** – Análise de correlação do intervalo QTc, intervalo Tp-e e ângulo QRS-T com outras variáveis

Variável	Intervalo QTc		Intervalo Tp-e		Ângulo QRS-T	

	r	p	r	p	r	p
Idade	0,204	0,045	0,309	0,002	0,396	<0,001
Índice de massa corporal	-0,001	0,990	-0,019	0,860	-0,014	0,896
Classe *NYHA*	0,022	0,831	0,051	0,657	0,020	0,846
Creatinina	0,149	0,165	0,272	0,010	0,389	<0,001
Taxa de filtração glomerular	-0,184	0,087	-0,294	0,005	-0,412	<0,001
FEVE	-0,328	0,001	0,028	0,788	0,341	0,001
DDVE	0,256	0,013	0,089	0,398	0,442	<0,001
DSVE	0,269	0,009	0,080	0,446	0,401	<0,001
Índice VDFVE	0,149	0,157	0,060	0,568	0,280	0,007
Índice VSFVE	0,171	0,103	-0,015	0,890	0,333	0,001
Número de segmentos do VE com RTG	0,262	0,009	0,564	<0,001	0,369	<0,001
Intervalo QTc			0,338	0,001	0,505	<0,001
Intervalo Tp-e	0,338	0,001			0,368	<0,001
Ângulo QRS-T	0,505	<0,001	0,368	<0,001		

*NYHA: New York Heart Association; FEVE: fração de ejeção do ventrículo esquerdo; DDFVE: diâmetro diastólico final do ventrículo esquerdo; DSFVE: diâmetro sistólico final do ventrículo esquerdo; VDFVE: volume diastólico final do ventrículo esquerdo; VSFVE: volume sistólico final do ventrículo esquerdo; VE: ventrículo esquerdo.*

A análise de regressão univariada foi realizada para determinar as variáveis preditoras do RTG por RMC em pacientes com ICFEr ( Tabela 4 ). Foram considerados significativos idade, sexo masculino, hiperlipidemia, doença arterial coronariana, intervalo QTc, intervalo Tp-e, ângulo QRS-T e nível de creatinina plasmática. Com essas variáveis quatro modelos diferentes foram gerados, sendo realizada a análise de regressão multivariada. No primeiro modelo, foram incluídas todas as três variáveis, intervalo QTc, intervalo Tp-e e ângulo QRS-T, e outras variáveis significativas ( Tabela 4 ). Em outros três modelos, esses parâmetros foram avaliados separadamente ( Tabela 5 ). No primeiro modelo, o sexo masculino (p = 0,032), doença arterial coronariana (p = 0,009), nível de creatinina plasmática (p = 0,037) e intervalo Tp-e [p = 0,001, OR (IC 95%) = 1,085 (1,032-1,140)] permaneceram significativos e foram preditores independentes da presença de RTG. Neste modelo, o intervalo QTc (p = 0,185) e o ângulo QRS-T (p = 0,944) perderam sua significância. No modelo que avaliou apenas o intervalo QTc, este intervalo (p=0,007) também foi um preditor independente, assim como sexo masculino, doença arterial coronariana e nível de creatinina plasmática. No modelo que avaliou apenas o intervalo Tp-e, ele permaneceu significativo (p <0,001) como no primeiro modelo. No modelo que avaliou apenas o ângulo QRS-T, o sexo masculino e a doença arterial coronariana foram preditores independentes, mas o ângulo QRS-T não alcançou significância (p=0,058).

**Tabela 4:** – Análise de regressão univariada e multivariada para determinar a predição de RTG em pacientes com IC

Variável	Análise univariada		Análise multivariada	

	OR (IC 95%)	p	OR (IC 95%)	p
Idade	1,051 (1,016-1,086)	0,004	0,960 (0,901-1,023)	0,209
Sexo masculino	11,936 (3,230-44,114)	<0,001	6,348 (1,172-34,392)	0,032
Índice de massa corporal	0,927 (0,831-1,034)	0,173		
Diabetes mellitus	1,501 (0,615-3,668)	0,373		
Hipertensão	0,958 (0,272-3,379)	0,947		
Hiperlipidemia	2,812 (1,224-6,464)	0,015	0,484 (0,105-2,226)	0,352
Doença arterial coronariana	8,036 (2,986-21,624)	<0,001	12,355 (1,851-82,445)	0,009
BRE	0,841 (0,288-2,459)	0,752		
Fibrilação atrial	0,490 (1,110-2,176)	0,348		
Duração do QRS	0,996 (0,979-1,013)	0,646		
Intervalo QTc	1,016 (1,006-1,027)	0,002	1,011 (0,995-1,028)	0,185
Intervalo Tp-e	1,099 (1,055-1,144)	<0,001	1,085 (1,032-1,140)	0,001
Ângulo QRS-T	1,017 (1,008-1,026)	<0,001	0,999 (0,984-1,015)	0,944
FEVE	0,976 (0,935-1,019)	0,273		
Índice VDFVE	1,003 (0,992-1,013)	0,637		
Índice VSFVE	1,003 (0,991-1,013)	0,657		
Creatinina	18,678 (3,460-100,82)	0,001	12,501 (1,170-133,63)	0,037

*OR: Odds ratio; IC: intervalo de confiança; BRE: bloqueio de ramo esquerdo; FEVE: fração de ejeção do ventrículo esquerdo; VDFVE: volume diastólico final do ventrículo esquerdo; VSFVE: volume sistólico final do ventrículo esquerdo.*

**Tabela 5:** – Análise de regressão multivariada para determinar o preditor independente de RTG em pacientes com IC

Variável	Análise multivariada					

	Intervalo QTc		Intervalo Tp-e		Ângulo QRS-T	

	OR (IC 95%)	p	OR (IC 95%)	p	OR (IC 95%)	p
Idade	0,974 (0,921-1,030)	0,359	0,964 (0,905-1,027)	0,252	0,980 (0,927-1,036)	0,469
Sexo masculino	6,393 (1,468-27,84)	0,013	7,405 (1,383-39,64)	0,019	7,369 (1,617-33,58)	0,010
Hiperlipidemia	0,643 (0,169-2,452)	0,518	0,513 (0,118-2,226)	0,626	0,727 (0,202-2,621)	0,398
Doença arterial coronariana	11,676 (2,43-56,09)	0,002	10,989 (1,89-63,69)	0,008	7,227 (1,605-32,55)	0,010
Creatinina	9,551 (1,548-58,92)	0,015	10,892 (1,26-94,01)	0,030	5,299 (0,947-29,66)	0,058
QTc, Tp-e, ângulo QRS-T	1,018 (1,005-1,032)	0,007	1,097 (1,045-1,152)	<0,001	1,010 (1,000-1,021)	0,058

*OR: odds ratio; IC: intervalo de confiança.*

**Figura 1:**
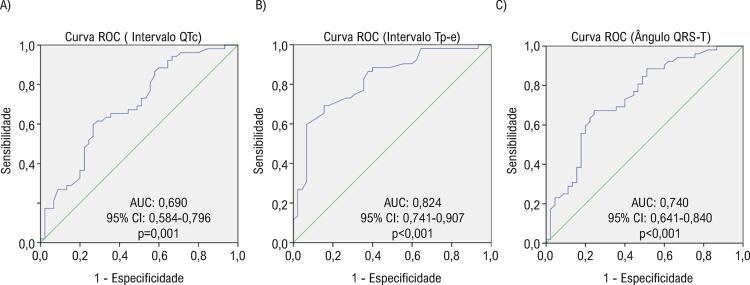
– Curva característica de operação do receptor mostrando a capacidade de diferenciação do intervalo QTc, intervalo Tp-e e ângulo QRS-T para a presença de RTG por RMC na ICFEr.

**Figura 2:**
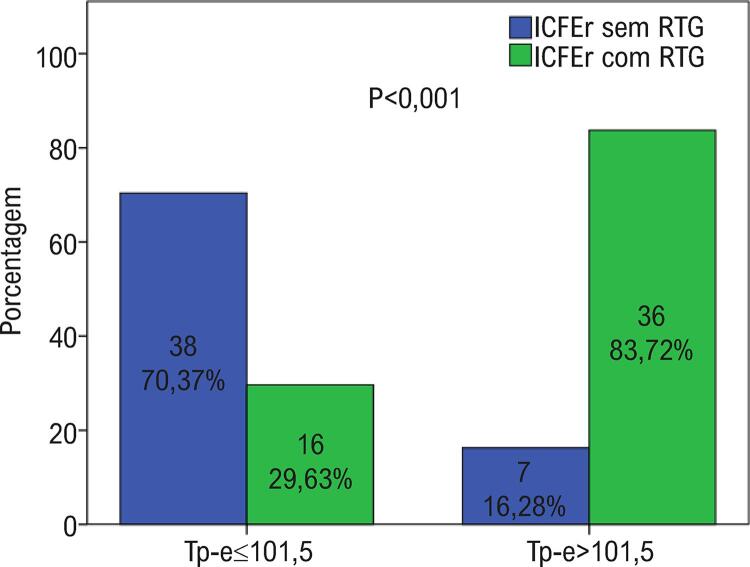
– Comparação das taxas de RTG nos grupos intervalo Tp-e baixo e alto. ICFEr: insuficiência cardíaca com fração de ejeção reduzida; RTG: realce tardio pelo gadolínio

Foram geradas curvas ROC para QTc, intervalo Tp-e e ângulo QRS-T para a presença de RTG por RMC em pacientes com ICFEr ( Figura 1 ). Embora haja necessidade de confirmação com estudos prospectivos, de acordo com as curvas ROC obtidas o melhor valor de corte do intervalo QTc foi de 460,5 ms, o valor de corte do intervalo Tp-e foi de 101,5 ms e o valor de corte do ângulo QRS-T foi 110 graus na identificação de pacientes com ICFEr com RTG. Quando os pacientes foram divididos em dois grupos de acordo com o ponto de corte de Tp-e, que apresentou o melhor valor de AUC, havia 54 pacientes (55,7%) no grupo Tp-e ≤101,5 e 43 pacientes (44,3%) no grupo Tp-e >101,5. No grupo Tp-e >101,5, os pacientes apresentaram razão RTG significativamente maiores do que o outro grupo ( Figura 2 ). De maneira similar a esses achados, os valores das medianas dos números do segmento RTG foram significativamente maiores [5,0 (3,0-9,0) vs. 0,0 (0,0-2,25), p <0,001] no grupo Tp-e >101,5.

## Discussão

Em nosso estudo, constatamos que o intervalo QTc, intervalo Tp-e e o ângulo QRS-T eram significativamente maiores em pacientes com ICFEr e RTG na RMC em comparação com pacientes com ICFEr sem RTG. Ainda na análise de correlação, verificou-se que os melhores coeficientes de correlação foram encontrados entre as medidas da RMC, que mostram a estrutura e função cardíacas, e o ângulo QRS-T, número de segmentos do ventrículo esquerdo com RTG e o intervalo Tp-e. Na análise de regressão multivariada, realizada por diferentes modelos, o intervalo Tp-e foi considerado o melhor parâmetro entre os três para predizer a presença de RTG em pacientes com ICFEr. Além disso, o intervalo Tp-e apresentou o maior valor de AUC na análise de curvas ROC realizada para a presença de RTG.

Os pacientes com IC foram divididos em três grupos de acordo com a FE na última diretriz da *European Society of Cardiology* (ESC) publicada em 2016. O grupo com FE inferior a 40% foi classificado como ICFEr, o grupo com FE de 50% ou mais foi classificado como IC com FE preservada (ICFEp) e o grupo com FE de 40–49% foi classificado como IC com FE intermediária (ICFEi). ^1^ As maiores taxas de mortalidade foram observadas no grupo ICFEr. ^21^ Uma das razões importantes de mortalidade são as arritmias malignas. A fibrose miocárdica, que é o substrato dessas arritmias, pode ser detectada com RTG por RMC. ^8^ Na literatura existem vários estudos que mostram a relação entre a presença de RTG e eventos cardiovasculares adversos em pacientes com IC. ^9 - 11 , 22^

Liu et al. mostraram a relação entre a quantidade de RTG detectado por RMC e eventos cardíacos adversos maiores (ECAM) em 84 pacientes com IC no estágio C ou D, isquêmicos ou não [p = 0,022, FC (IC 95%) = 1,045 (1,001-1,084)]. ^11^ Shi et al., ^22^ declararam, em sua meta-análise que continha cinco estudos e 545 pacientes com cardiomiopatia dilatada (CMD), que a presença de RTG foi o preditor de alta mortalidade cardiovascular [p = 0,03, OR (IC 95%) = 2,67 (1,12-6,35)], MSC abortada [p = 0,007, OR (IC 95%) = 5,26 (1,57-17,55)] e hospitalização devido a IC [p <0,001, OR (IC 95%) = 3,91 (1,99-7,69)]. ^22^ Na meta-análise de Duan et al., ^9^ que avaliaram 13 estudos e incluíram 1.675 pacientes com CMD, foram investigados os efeitos da presença de RTG sobre os ECAM. Nestes 13 estudos, as taxas de presença de RTG situaram-se entre 18% e 71%. Os resultados da análise mostraram que o RTG estava associado a todas as causas de mortalidade [p <0,001, OR (IC 95%) = 3,43 (2,26-5,22)], morte cardíaca [p <0,001, OR (IC 95%) = 3,65 (1,80- 7,40)], hospitalização por IC [p = 0,001, OR (IC 95%) = 2,87 (1,53-5,39)] e eventos arrítmicos maiores [p <0,001, OR (IC 95%) = 4,24 (2,95-6,08)] e MSC [p <0,001, OR (IC 95%) = 3,33 (1,80-6,17)]. ^9^

Embora a RMC forneça informações importantes aos médicos, ela tem algumas limitações. A RMC não está amplamente disponível, as interpretações da RMC precisam de especialização, os custos são altos e a sua segurança para pacientes com implantes metálicos ainda é debatida, podendo ser menos confiável em pacientes taquiarrítmicos e claustrofóbicos; o uso de gadolínio como agente de contraste em pacientes com insuficiência renal grave é outra limitação importante. Portanto, pode ser de grande conveniência prever a fibrose miocárdica com parâmetros de repolarização calculados a partir do ECG, os quais são facilmente disponíveis e interpretáveis. O presente estudo foi realizado com esse objetivo e investigamos se o intervalo QTc, o intervalo Tp-e e o ângulo QRS-T frontal estão relacionados com a presença de RTG e qual desses parâmetros pode ser o melhor preditor.

Os intervalos QT e QTc são parâmetros e bem conhecidos e amplamente utilizados da repolarização miocárdica estão relacionados à arritmia ventricular e mortalidade cardiovascular. ^23^ Em alguns estudos é demonstrado que a duração entre o pico e o ponto final da onda T (intervalo Tp-e) é um novo marcador para avaliar a repolarização ventricular, e por não ser afetado pela variabilidade da frequência cardíaca, o intervalo Tp-e pode ser mais confiável para avaliar a repolarização ventricular. Além disso, as relações Tp-e/QT e Tp-e/QTc demonstraram ser um índice sensível de repolarização e arritmogênese ventricular, pois fornecem uma estimativa da dispersão da repolarização em relação à duração total da repolarização. ^24 , 25^ Outro novo marcador é o ângulo QRS-T espacial. Ele é definido como a diferença angular entre a direção da despolarização ventricular (onda QRS) e a direção da repolarização ventricular (onda T). ^18^ Porém, o cálculo do ângulo QRS espacial é muito complicado e requer programação de computador avançada. ^26^ Por outro lado, o ângulo QRS frontal pode ser medido facilmente a partir de laudos automáticos de aparelhos de ECG e tem uma boa correlação com o ângulo QRS espacial na estratificação de risco. ^27^ Por este motivo utilizamos o ângulo QRS-T frontal em nosso estudo.

Estudos anteriores revelaram que em pacientes com IC, os parâmetros de repolarização estavam associados a mortalidade por todas as causas, morte cardíaca, hospitalização por IC, MSC e choques adequados em pacientes com cardioversor-desfibrilador implantável (CDI). ^28 - 31^

Que seja de nosso conhecimento, nenhum estudo investigou a relação do RTG com RMC e os parâmetros de repolarização, e, portanto, buscamos atender esse déficit. Em nosso estudo, semelhante aos da literatura, o índice de RTG foi de 53,6% em pacientes com ICFEr. Todos os parâmetros de repolarização foram significativamente maiores em pacientes com RTG. Na análise univariada, todos os três parâmetros previram a presença de RTG separadamente. Na análise multivariada, primeiro foi gerado um modelo que incluiu todos os três parâmetros de repolarização e as outras variáveis significativas. Neste modelo, apenas o intervalo Tp-e, dentre os parâmetros de repolarização, permaneceu significativo e foi considerado um preditor independente da presença de RTG [p=0,001, OR (IC 95%) = 1,085 (1,032-1,140)]. A partir de um ponto de vista diferente, ao inserirmos os parâmetros separadamente nos modelos, o intervalo QTc e o intervalo Tp-e permaneceram significativos, mas o nível de significância do intervalo Tp-e foi melhor do que o do intervalo QTc (p <0,001 vs. p=0,007). Por outro lado, o intervalo Tp-e apresentou correlação média (r = 0,564, p<0,001) com o número de segmentos do VE com RTG, enquanto os outros dois parâmetros apresentaram correlações fracas. Como resultado, em nosso estudo, o intervalo Tp-e mostrou-se mais relacionado com o RTG em pacientes com ICFEr quando comparado aos outros dois parâmetros de repolarização. A estimativa do risco de ECAM em pacientes com ICFEr pode ser possível com este parâmetro, o qual pode ser facilmente medido a partir do ECG padrão de 12 derivações.

Nosso estudo apresentou algumas limitações. Em primeiro lugar, o desenho do estudo foi retrospectivo, de centro único, e com uma população de estudo pequena. Nenhum voluntário saudável foi incluído no estudo. Não houve dados de seguimento clínico. Pacientes com IC foram incluídos no estudo independentemente da etiologia. A avaliação dos pacientes separadamente de acordo com a etiologia isquêmica ou não-isquêmica poderia fornecer mais informações. Os dados da RMC são limitados em nosso estudo. O RTG é fornecido apenas como presença ou ausência e o número de segmentos que mostram envolvimento. A disponibilidade de dados adicionais mostrando a transmuralidade da cicatriz, porcentagem de cicatriz do VE e *strain* regional poderiam enriquecer o estudo. Finalmente, neste estudo, os valores do ponto de corte dos parâmetros de repolarização foram desenvolvidos utilizando curvas ROC. Portanto, nossos resultados devem ser interpretados com cautela até que sejam confirmados em estudos subsequentes.

## Conclusão

Em nosso estudo, encontramos um intervalo QTc, intervalo Tp-e e ângulo QRS-T frontal mais elevados em pacientes com ICFEr e RTG por RMC quando comparados a pacientes com ICFEr sem RTG. O intervalo Tp-e foi o melhor preditor independente da presença de RTG. Essa informação, com o prolongamento do intervalo Tp-e, permite prever a presença de fibrose miocárdica, que é um substrato arritmogênico, em pacientes com ICFEr. Como resultado, acreditamos que com um ECG padrão de 12 derivações, facilmente disponível e interpretado, pode ser possível prever o risco de eventos cardiovasculares adversos em pacientes com ICFEr; também acreditamos que a redução nas taxas de ECAM pode ser obtida com terapia médica intensiva, seguimento rigoroso e terapia com CDI. Sabemos que mais estudos prospectivos, randomizados e com uma população maior são necessários para apoiar nossas opiniões.

## References

[1] Ponikowski P , Voors AA , Anker SD , Bueno H , Cleland JG , Coats AJ , et al ( 2016 ). ESC Guidelines for the diagnosis and treatment of acute and chronic heart failure: The Task Force for the diagnosis and treatment of acute and chronic heart failure of the European Society of Cardiology (ESC). Developed with the special contribution of the Heart Failure Association (HFA) of the ESC . Eur J Heart Fail . 2016 ; 18 ( 8 ), 891 - 975 . 10.1002/ejhf.592 27207191

[2] Yancy CW , Jessup M , Bozkurt B , Butler J , Casey DE , Colvin MM , et al . 2017 ACC/AHA/HFSA focused update of the 2013 ACCF/AHA guideline for the management of heart failure: a report of the American College of Cardiology/American Heart Association Task Force on Clinical Practice Guidelines and the Heart Failure Society of America . J Am Coll Cardiol . 2017 ; 70 ( 6 ), 776 - 803 . 10.1016/j.jacc.2017.04.025 28461007

[3] Goldstein S , Hjalmarson A . The mortality effect of metoprolol CR/XL in patients with heart failure: results of the MERIT-HF Trial . Clin Cardiol . 1999 ; *22* , V30 - 5 . 10526701

[4] Narang R , Cleland JGF , Erhardt L , Ball SG , Coats , AJS , Cowley AJ , et al . Mode of death in chronic heart failure: a request and proposition for more accurate classification . Eur Heart J . 1996 ; *17* ( 9 ), 1390 - 403 . 10.1093/oxfordjournals.eurheartj.a015074 8880025

[5] Bernardo BC , Weeks KL , Pretorius L , McMullen JR . Molecular distinction between physiological and pathological cardiac hypertrophy: experimental findings and therapeutic strategies . Pharmacol Therap . 2010 ; 128 ( 1 ), 191 - 227 . 10.1016/j.pharmthera.2010.04.005 20438756

[6] Piek A , De Boer RA , Silljé HHW . The fibrosis-cell death axis in heart failure . Heart Fail Rev . 2016 ; 21 ( 2 ), 199 - 211 . 10.1007/s10741-016-9536-9 PMC4762920 26883434

[7] Weeks KL , McMullen JR . The athlete’s heart vs. the failing heart: can signaling explain the two distinct outcomes? . Physiology . 2011 ; *26* ( 2 ), 97 - 105 . 10.1152/physiol.00043.2010 21487028

[8] Nanjo S , Yoshikawa K , Harada M , Inoue Y , Namiki A , Nakano H , et al . Correlation between left ventricular diastolic function and ejection fraction in dilated cardiomyopathy using magnetic resonance imaging with late gadolinium enhancement . Circulation . 2009 ; 73 ( 10 ), 1939 - 44 . 10.1253/circj.cj-08-0965 19729860

[9] Duan , X , Li J , Zhang Q , Zeng Z , Luo Y , Jiang J , et al . Prognostic value of late gadolinium enhancement in dilated cardiomyopathy patients: a meta-analysis . Clin Radiol . 2015 ; 70 ( 9 ), 999 - 1008 . 10.1016/j.crad.2015.05.007 26116301

[10] Becker MA , Cornel JH , Van de Ven PM , van Rossum AC , Allaart CP , Germans , T . The prognostic value of late gadolinium-enhanced cardiac magnetic resonance imaging in nonischemic dilated cardiomyopathy: a review and meta-analysis . JACC: Cardiovasc Imag . 2019 ; 11 ( 9 ), 1274 - 84 . 10.1016/j.jcmg.2018.03.006 29680351

[11] Liu T , Ma X , Liu W , Ling S , Zhao L , Xu L , et al . Late gadolinium enhancement amount as an independent risk factor for the incidence of adverse cardiovascular events in patients with stage C or D heart failure . Front Physiol . 2016 ; *7* , 484 . 10.3389/fphys.2016.00484 PMC5083842 27840608

[12] Taşolar H , Ballı M , Çetin M , Otlu YÖ , Altun B , Bayramoğlu A . Effects of the Coronary Collateral Circulation on the Tp-e Interval and Tp-e/QT Ratio in Patients with Stable Coronary Artery Disease . Ann Noninvas Electrocard 2015 ; *20* ( 1 ), 53 - 61 . 10.1111/anec.12173 PMC6931662 24934391

[13] Kors JA , van Eck HJR , van Herpen G . The meaning of the Tp-Te interval and its diagnostic value . J Electrocard . 2008 ; *41* ( 6 ), 575 - 80 . 10.1016/j.jelectrocard.2008.07.030 18954608

[14] Aro AL , Huikuri HV , Tikkanen JT , Junttila MJ , Rissanen HA , Reunanen A , et al . QRS-T angle as a predictor of sudden cardiac death in a middle-aged general population . Europace . 2011 ; 14 ( 6 ), 872 - 6 . 10.1093/europace/eur393 22183749

[15] Chua KC , Teodorescu C , Reinier K , Uy-Evanado A , Aro AL , Nair SG , et al . Wide QRS-T Angle on the 12-Lead ECG as a Predictor of Sudden Death Beyond the LV Ejection Fraction . J Cardiovasc Electrophys . 2016 ; 27 ( 7 ), 833 - 9 . 10.1111/jce.12989 PMC5039018 27094232

[16] Borleffs CJW , Scherptong RW , Man SC , van Welsenes GH , Bax JJ , van Erven L , et al . Predicting ventricular arrhythmias in patients with ischemic heart disease: clinical application of the ECG-derived QRS-T angle . Circulation: Arrhyth Electrophysiol . 2009 ; 2 ( 5 ), 548 - 54 . 10.1161/CIRCEP.109.859108 19843923

[17] Wang B , Zhang LI , Cong P , Chu H , Liu Y , Liu J , et al . A new formula for estimating the true QT interval in left bundle branch block . J Cardiovasc Electrophys . 2017 ; 28 ( 6 ), 684 - 9 . 10.1111/jce.13203 28297125

[18] Oehler A , Feldman T , Henrikson CA , Tereshchenko LG . QRS-T angle: a review . Ann Noninvas Electr . 2014 ; 19 ( 6 ), 534 - 42 . 10.1111/anec.12206 PMC4237708 25201032

[19] Kramer CM , Barkhausen J , Flamm SD , Kim RJ , Nagel E . Standardized cardiovascular magnetic resonance imaging (CMR) protocols, society for cardiovascular magnetic resonance: board of trustees task force on standardized protocols . J Cardiovasc Magn Reson . 2008 ; 10 ( 1 ), 35 . 10.1186/1532-429X-10-35 PMC2467420 18605997

[20] Simonetti OP , Kim RJ , Fieno DS , Hillenbrand HB , Wu E , Bundy JM , et al . An improved MR imaging technique for the visualization of myocardial infarction . Radiology . 2001 ; 218 ( 1 ), 215 - 23 . 10.1148/radiology.218.1.r01ja50215 11152805

[21] Lam CS , Solomon SD . The middle child in heart failure: heart failure with mid-range ejection fraction (40–50%) . Eur Heart J . 2014 ; 16 ( 10 ), 1049 - 55 . 10.1002/ejhf.159 25210008

[22] Shi HW , Pu P , Deng W , Zhou H , Bian ZY , Shen DF , et al . Prognostic value of late gadolinium enhancement in dilated cardiomyopathy patients. A meta-analysis . Saudi Med J . 2016 ; 34 ( 7 ), 719 . 23860892

[23] Algra A , Tijssen JG , Roelandt JR , Pool J , Lubsen J . QTc prolongation measured by standard 12-lead electrocardiography is an independent risk factor for sudden death due to cardiac arrest . Circulation . 1991 ; 83 ( 6 ), 1888 - 94 . 10.1161/01.cir.83.6.1888 2040041

[24] Antzelevitch C , Sicouri S , Di Diego JM , Burashnikov A , Viskin S , Shimizu W , et al . Does Tpeak–Tend provide an index of transmural dispersion of repolarization? . Heart Rhythm . 2007 ; 4 ( 8 ), 1114 - 6 . 10.1016/j.hrthm.2007.05.028 PMC1994816 17675094

[25] Gupta P , Patel C , Patel H , Narayanaswamy S , Malhotra B , Green JT , et al . Tp-e/QT ratio as an index of arrhythmogenesis . Journal of electrocardiology . 2008 ; 41 ( 6 ), 567 - 574 . 10.1016/j.jelectrocard.2008.07.016 18790499

[26] Okin PM . Electrocardiography in women: taking the initiative . Circulation . 2006 ; 113 ( 4 ): 464 - 6 . 10.1161/CIRCULATIONAHA.105.581942 16449723

[27] Zhang ZM , Prineas RJ , Case D , Soliman EZ , Rautaharju PM , ARIC Research Group . Comparison of the prognostic significance of the electrocardiographic QRS/T angles in predicting incident coronary heart disease and total mortality (from the atherosclerosis risk in communities study) . Am J Cardiol . 2007 ; 100 ( 5 ), 844 - 9 . 10.1016/j.amjcard.2007.03.104 PMC2238030 17719331

[28] Zhu TY , Teng SE , Chen YY , Liu SR , Meng SR , Peng J . Correlation of Tp-e interval and Tp-e/QT ratio with malignant ventricular arrhythmia in patients with implantable cardioverter-defibrillator for primary prevention. Nan fang yi ke da xue xue bao= J South Med Univ . 2016 ; 36 ( 3 ), 401 - 4 . 27063171

[29] Sen Ö , Yilmaz S , Sen F , Balcı KG , Akboga MK , Yayla C , et al . T-peak to T-end Interval Predicts Appropriate Shocks in Patients with Heart Failure Undergoing Implantable Cardioverter Defibrillator Implantation for Primary Prophylaxis . Ann Noninvasa Electrol . 2016 . 10.1111/anec.12383 PMC10825066 27265779

[30] 30 Gotsman I , Shauer A , Elizur Y , Zwas DR , Lotan C , Keren A . Temporal changes in electrocardiographic frontal QRS-T angle and survival in patients with heart failure . PloS One . 2018 ; 13 ( 3 ): e0194520 . 10.1371/journal.pone.0194520 PMC5868814 29579123

[31] Li SN , Zhang XL , Cai GL , Lin RW , Jiang H , Chen JZ , et al . Prognostic sMed J. Significance of frontal QRS-T angle in patients with idiopathic dilated cardiomyopathy . Chin . 2016 ; 129 ( 16 ): 1904 . 10.4103/0366-6999.187844 PMC4989419 27503013

