# Supplementary material for: Relação entre o Realce Tardio pelo Gadolínio e os Parâmetros de Repolarização Ventricular em Pacientes com Insuficiência Cardíaca com Fração de Ejeção Reduzida
Source: Arq Bras Cardiol. 2021 Jun 25;117(4):678–87. [Article in Portuguese] doi: 10.36660/abc.20200149 (PMC8528364; doi:10.36660/abc.20200149)
Supplement: Translation

# Translation: Relationship between Late Gadolinium Enhancement and Ventricular Repolarization Parameters in Heart Failure Patients with Reduced Ejection Fraction

**Authors:** Ali Riza Demir, Omer Celik, Songül Ustündağ, Begum Uygur, Mustafa Umut Somuncu, Emre Yilmaz, Yalcin Avci, Gokhan Demirci, Serkan Kahraman, Mehmet Erturk

**Affiliations:** 1 Turkey Cardiology Department, University of Health Science, Istanbul Mehmet Akif Ersoy Thoracic and Cardiovascular Surgery Training and Research Hospital, Istanbul - Turkey; 2 Turkey Cardiology Department, Erzincan Binali Yıldırım University Mengücek Gazi Training and Research Hospital, Erzincan - Turkey; 3 Turkey Cardiology Department, Zonguldak Bülent Ecevit University Faculty of Medicine, Zonguldak - Turkey; 4 Turkey Cardiology Department, Görele Op. Dr. Ergun Ozdemir State Hospital, Giresun - Turkey

**Keywords:** Heart Failure, Gadolinium, Stroke Volume, Diagnostic, Imaging, Magnetic Resonance Spectroscopy, Electrocardiography/methods, Continuity of Patient Care/ethic

## Abstract

**Background:**

Heart failure with reduced ejection fraction (HFrEF) is a highly prevalent disease that requires repeating hospitalizations, causes significant morbidity and mortality. Therefore, early recognition of poor outcome predictors is essential for patient management.

**Objective:**

The aim of the present study is to investigate the relationship between late gadolinium enhancement (LGE) detected by cardiac magnetic resonance (CMR) and repolarization parameters such as corrected QT (QTc) interval, Tp-e interval, frontal QRS-T angle detected by 12 lead electrocardiograph (ECG) in HFrEF.

**Method:**

In this single-center, retrospective observational study included 97 consecutive HFrEF patients who had CMR scan. Study population was divided into two groups according to the presence of LGE. Echocardiographic and CMR measurements and demographic features were recorded. QTc intervals, Tp-e intervals, frontal QRS-T angles were calculated from the ECG. A p-value less than 0.05 was considered statistically significant.

**Results:**

LGE was detected in 52 (53.6%) out of 97 HFrEF patients. QTc intervals (p=0.001), Tp-e intervals (p<0.001), frontal QRS-T angles (p<0.001) were found to be significantly higher in LGE group when compared to non-LGE group. In univariate regression analysis which was performed to investigate the predictors of LGE in HFrEF, all three repolarization parameters were reached significant values but in multivariate analysis the only repolarization parameter remained significant was Tp-e interval (OR=1.085 95% CI 1.032-1.140, p=0.001).

**Conclusion:**

With the prolongation of the Tp-e interval, the presence of myocardial fibrosis which is an arrhythmogenic substrate, can be predicted in patients with HFrEF.

## Introduction

Heart failure (HF) is a highly prevalent disease in the general population that requires repeat hospitalizations and causes significant morbidity and mortality.^1 , 2^ As a consequence of these serious outcomes, HF results in a high economic burden for healthcare systems. Therefore, early recognition of poor outcome predictors is essential for patient management. Mortality occurs commonly due to pump failure or arrhythmogenic episodes.^3 , 4^ Whether ischemic or nonischemic origin, patients with HF have more myocardial fibrosis than healthy subjects which is a substrate for arrhythmias and negative remodeling which gradually decreases left ventricular functions.^5 - 7^

Late gadolinium enhancement (LGE) from cardiac magnetic resonance (CMR) is capable of detecting tissue abnormalities, particularly myocardial fibrosis.^8^ Researchers recently discovered that, in addition to LVEF, LGE could be used as a marker of poor prognosis in patients with HF.^9 - 11^

In clinical practices, ECG is widely used to predict the arrhythmogenic risk. QTc interval, Tp-e interval and QRS-T angle are ventricular repolarization parameters, recommended as predictors of malignant ventricular arrhythmias. Tp-e is a relatively new parameter for transmural repolarization dispersion and also related with the sudden cardiac death (SCD) risk.^12 , 13^ Frontal QRS-T angle defined as an angle between ventricular depolarization and repolarization directions, reflects electrical heterogeneity and abnormal changes in direction of repolarization sequence due to cardiac structural abnormalities and frontal QRS-T angle is a strong predictor of electrical instability and SCD.^14 - 16^

We aimed to investigate the relationship between LGE detected by CMR and repolarization parameters such as QTc interval, Tp-e interval, frontal QRS-T angle detected by 12 lead ECG inHFrEF patients and to determine which one of these parameters is more significant in this aspect.

## Methods

### Study population

This study included 97 HFrEF patients admitted to the cardiology clinic between January 2017 and June 2019, who had undergone CMR for various reasons (HF etiology research, viability research, EF calculation, etc.) and had an EF <40% on transthoracic echocardiography.

Data regarding patients’ demographic characteristics (age, gender and body mass index), medical history [diabetes mellitus (DM), hypertension (HT), hyperlipidemia (HL) and coronary artery disease], medications [beta-blockers, angiotensin-converting enzyme inhibitors (ACEi), angiotensin receptor blockers (ARB), spironolactone, diuretics, digoxin, statins], cardiac rhythm [sinus rhythm, atrial fibrillation, and left bundle branch block (LBBB)], echocardiographic examination results, 12-lead ECG and biochemical blood tests were recorded. The NYHA functional class of each patient was determined.

Patients who were aged <18 years or >90 years; who had a history of acute coronary syndrome or primary coronary intervention within the past 6 months; and who had hypotension, pulmonary edema, or cardiogenic shock were excluded from the study. In addition, patients with stage 4-5 chronic kidney disease (CKD); those with an active focus of infection, neurological illness severe enough to affect biochemical and hematological results, chronic obstructive pulmonary disease (COPD), malignancy or liver function impairment/liver failure were excluded.

The study was approved by the ethics committee of Mehmet Akif Ersoy Thoracic and Cardiovascular Surgery Training Research Hospital. This study was performed in accordance with the requirements of the Declaration of Helsinki.

### Electrocardiography

12-lead ECG was performed at paper speed of 25 mm/second with the patient at rest in supine position. Resting heart rate was then measured from ECG data. All ECG data were scanned and transferred to personal computer and used for x400% magnification with Adobe Photoshop software (Adobe Systems, Inc., San Jose, CA, USA). ECG measurements of QTc, Tp-e intervals and frontal QRS-T angle were performed by 2 cardiologists who were blinded to the patient's CMR data. Subjects with U wave on their ECG were excluded from the study. Average value of 3 examinations was calculated for each lead. QT interval was measured from beginning of QRS complex to end of T wave. In patients with LBBB, the formula [QT = QTLBBB - (0.86 x QRSLBBB - 71)] recommended by Wang et al was used^17^ and QT interval was corrected by heart rate using Bazett’s formula [QTc=QT√ (R–R interval)]. Tp-e interval was defined as interval from peak of T wave to end of T wave. Measurements of Tp-e interval were performed from precordial leads. Tp–e/QT ratio and Tp-e/QTc ratio were calculated from these measurements. The frontal QRS-T angle was calculated as the absolute value of the difference between the frontal plane QRS and T axes. If such a difference was more than 180°, QRS-T angle was adjusted to the minimal angle as 360° minus the absolute value of the difference between the frontal plane QRS and T axes.^18^ The intra-researcher and inter-researcher differences for QTc, Tp-e intervals and QRS-T angle were less than 5%.

### Image acquisition

Images were acquired using 1.5 T scanners (MAGNETON Aera, Siemens, Erlangen, Germany) with full myocardial coverage. Balanced steady-state free-precession sequences were used to obtain breath-hold cine images in three long-axis planes, followed by a contiguous stack of short-axis slices from the atrioventricular ring to the apex.^19^ Late enhancement images were acquired 10 min after the administration of 0.1 mmol/kg intravenous gadolinium contrast agent (gadopentetate dimeglumine/gadobutrol, Bayer, Berlin, Germany) with an inversion recovery-prepared gradient-echo sequence.^20^ Inversion times were optimised to null normal myocardium with images acquired in two orthogonal phase-encoding directions to exclude artefact.

### Image analysis

Images were transferred to a workstation (Leonardo, Siemens Medical Solutions, Erlangen, Germany) for analysis. For the functional analysis, commercially available software program, Argus (Siemens Healthcare, Erlangen, Germany) was used.

### CMR data analysis

The endocardial and epicardial borders were traced manually using both software systems and functional analysis was performed.

Analysis using Argus (Siemens Healthcare, Erlangen, Germany) Software for each study, the end-diastolic and end-systolic phases were determined. For the detection of each phase, the largest and narrowest diameters of the ventricular cavity at the middle of the ventricle were used. The endocardial and epicardial borders were traced manually in short axis images in both phases. The borders of the endocardium were traced by using the intensity difference between the chamber when filled with blood and the moderate intensity of the myocardium. The papillary muscles were included in the LV volumetric analysis. While the epicardial border was being detected, the interventricular septum was included in the LV volume. The most basal slice that was surrounded by at least 50% of the myocardium with filled blood was defined as the basal segment of the left ventricle. This was included in the LV chamber volume. The apex was defined as the last slice with a visible lumen throughout the entire cardiac cycle. The end-systolic volume (ESV), end-diastolic volume (EDV) and EF were determined according to the Simpson’s rule. The elapsed time from inputting of the data to obtaining the results was calculated for each patient.

CMR images were reanalyzed and documented using the 17-segment cardiac model recommended by the American Heart Association to improve standardization of the results. The left ventricle was evaluated from the short-axis images from basal, mid, and apical segments. The basal and mid cavity were divided into 6 equal segments: anterior, anteroseptal, inferoseptal, inferior, inferolateral, and anterolateral. The apical segment was divided into 4 segments: anterior, septal, inferior, and lateral. The apical cap was termed the apex and constituted the 17th segment. The contrast-enhanced images were analyzed visually by two experienced observers who were blinded to other MRI, echocardiographic, and clinical data. Late gadolinium enhancement was rated by visual assessment, and each segment was graded on a 2-point scale (segmental fibrosis score; 0 = absence of late gadolinium enhancement, 1 = presence of late gadolinium enhancement), using the method of Kaandorp et al. because of the frequency of linear and patchy enhancement in patients with nonischemic dilated cardiomyopathy.

## Statistical analysis

Data was analyzed using the Statistical Package for the Social Sciences, version 24.0 (SPSS Inc., Chicago, Illinois, USA). Whether the variables show normal distribution; was evaluated using visual (histograms, probability curves) and analytical (Kolmogorov-Smirnov) methods. Continuous variables showing normal distribution were mean ± standard deviation (SD), continuous variables not showing normal distribution were expressed as median (interquartile range) and categorical variables as percentage (%). Continuous variables such as QTc, Tp-e interval and QRS-T angle were evaluated using unpaired Student’s t-test and the Mann-Whitney U-test between the two groups. Chi-Square or Fisher exact test were used to compare categorical variables. The correlation between QTc, Tp-e interval and QRS-T angle and the other continuous variables were identified using Pearson or Spearman tests. Logistic regression analysis was performed to the determine the independent predictors of LGE presence with CMR in HFrEF patients. Firstly, univariate logistic regression analysis was performed, parameters which were significant in this analysis (p <0.05) were included in the multivariate logistic regression analysis. Receiver operator characteristic (ROC) curve analysis was carried out to determine the cutoff values. Significant prediction was accepted when the area under the ROC curve was more than 0.5; p<0.05 was accepted as statistically significant.

## Results

The mean age of the 97 HFrEF patients was 54.8±13.8. Our study population included 75 (77.3%) male and 22 (22.7%) female patients. We divided our patients into two groups according to the presence of LGE with CMR. LGE was detected in 52 (53.6%) patients. Basal demographics and laboratory results for both groups were in Table 1 .

**Table 1:** – Baseline demographic, clinical, and laboratory characteristics of the study groups

Variable	HFrEF without LGE (n=45)	HFrEF with LGE (n=52)	p-value
Age, years	50.2±15.6	58.7±10.7	0.002
Male, n (%)	26 (57.8%)	49 (94.2%)	<0.001
Body mass index, kg/m^2^	27.7±4.2	26.6±3.7	0.171
Diabetes mellitus, n (%)	11 (24.4%)	17 (32,7%)	0.371
Hypertension, n (%)	40 (88.9%)	46 (88.5%)	0.947
Coronary artery disease, n (%)	20 (44.4%)	45 (86.5%)	<0.001
Hyperlipidemia, n (%)	20 (44.4%)	36 (69.2%)	0.014
Heart rate, bpm	78.2±18.9	76.8±16.1	0.691
Atrial fibrillation, n (%)	5 (11.1%)	3 (5.8%)	0.466
LBBB, n (%)	8 (17.8)	8 (15.4)	0.751
NYHA class I, n (%)	10 (22.2%)	9 (17.3%)	0.543
NYHA class II, n (%)	32 (71.1%)	36 (69.2%)	0.840
NYHA class III, n (%)	3 (6.7%)	7 (13.5%)	0.331
Creatinine, mg/dL	0.80 (0.70-0.90)	1.10 (0.80-1.40)	<0.001
Glomerular filtration rate, ml/dk	91.3±25.1	71.9±26.3	0.001
Beta blockers, n (%)	42 (93.3%)	50 (96.2%)	0.661
ACEi or ARB, n (%)	40 (88.9%)	42 (80.8%)	0.270
Statins, n (%)	16 (35.6%)	36 (69.2%)	0.001
Diuretics, n (%)	25 (55.6%)	31 (59.6%)	0.686
Spironolactone, n (%)	31 (68.9%)	29 (55.8%)	0.185
Digoxin, n (%)	5 (11.1%)	2 (3.8%)	0.244
Diltiazem, n (%)	3 (6.7%)	2 (3.8%)	0.661
Verapamil, n (%)	0 (0.0%)	1 (1.9%)	1.0

*Data are presented as percentage, mean ± standard deviation or median (interquartile range). HFrEF: heart failure with reduced ejection fraction; LGE: late gadolinium enhancement; LBBB: left bundle branch block; NYHA: New York Heart Association; ACEi: angiotensin-converting enzyme inhibitor; ARB: angiotensin receptor blocker.*

Comparison of parameters calculated from ECG and variables detected by CMR were shown in Table 2 . Reflection of repolarization on ECG such as QT, QTc, Tp-e interval, Tp-e/QTc ratio and QRS-T angle were significantly higher in LGE group. In LGE group, mean 6.48±3.54 enhanced segments were detected.

**Table 2:** – Comparison of study groups in terms of some parameters calculated from CMR and ECG

Variable	HFrEF without LGE (n=45)	HFrEF with LGE (n=52)	p-value
QRS duration, ms	103.6±26.2	101.4±20.9	0.650
QT interval, ms	391.6±53.3	418.9±50.3	0.011
QTc interval, Bazett	438.2±42.3	467.3±42.9	0.001
Tp-e interval, ms	91.4±12.3	108.3±14.6	<0.001
Tp–e/QT ratio	0.235±0.034	0.261±0.042	0.001
Tp–e/QTc ratio	0.208±0.025	0.233±0.038	<0.001
QRS-T angle, degree	61 (28-112)	136 (82-159)	<0.001
LVEF, %	30.9±10.7	28.8±8.3	0.275
LVEDD, mm	57.3±8.8	57.7±7.7	0.826
LVESD, mm	45.9±10.5	45.6±8.7	0.891
LVEDV index, mL/m^2^	110.1±40.8	113.9±36.7	0.641
LVESV index, mL/m^2^	73.8±40.1	77.1±31.1	0.661
Number of LV segments with LGE		6.48±3.54	

*Data are presented as percentage, mean ± standard deviation or median (interquartile range). HFrEF: heart failure with reduced ejection fraction; LGE: late gadolinium enhancement; LVEF: left ventricular ejection fraction; LVEDD: left ventricular end diastolic diameter; LVESD: left ventricular end systolic diameter; LVEDV: left ventricular end diastolic volume; LVESV: left ventricular end systolic volume; LV: left ventricle.*

The correlation analysis of QTc, Tp-e interval and QRS-T angle with other variables was shown in Table 3 . There were poor correlations between QTc interval and LVEF, LVEDD and LVESD and also there were poor correlations between QRS-T angle and LVEF, LVEDD, LVESD, LVEDV index and LVESV index. Although there wasn't any correlation between Tp-e and CMR parameters show cardiac structures and functions, the best correlation coefficient between LGE segment number was obtained with Tp-e interval. There were medium correlation with Tp-e interval (r=0.564, p<0.001) and poor correlations with QTc interval (r=0.262, p=0.009) and QRS-T angle (r=0.369, p<0.001). Another finding of our study, there were various degrees of correlations between QTc, Tp-e interval, QRS-T angle themselves.

**Table 3:** – Correlation analysis of QTc, Tp-e interval and QRS-T angle with other variables

Variable	QTc interval		Tp-e interval		QRS-T angle	

	r	p	r	p	r	p
Age	0.204	0.045	0.309	0.002	0.396	<0.001
Body mass index	-0.001	0.990	-0.019	0.860	-0.014	0.896
NYHA class	0.022	0.831	0.051	0.657	0.020	0.846
Creatinine	0.149	0.165	0.272	0.010	0.389	<0.001
Glomerular filtration rate	-0.184	0.087	-0.294	0.005	-0.412	<0.001
LVEF	-0.328	0.001	0.028	0.788	0.341	0.001
LVEDD	0.256	0.013	0.089	0.398	0.442	<0.001
LVESD	0.269	0.009	0.080	0.446	0.401	<0.001
LVEDV index	0.149	0.157	0.060	0.568	0.280	0.007
LVESV index	0.171	0.103	-0.015	0.890	0.333	0.001
Number of LV segments with LGE	0.262	0.009	0.564	<0.001	0.369	<0.001
QTc interval			0.338	0.001	0.505	<0.001
Tp-e interval	0.338	0.001			0.368	<0.001
QRS-T angle	0.505	<0.001	0.368	<0.001		

*NYHA: New York Heart Association; LVEF: left ventricular ejection fraction; LVEDD: left ventricular end diastolic diameter; LVESD: left ventricular end systolic diameter; LVEDV: left ventricular end diastolic volume; LVESV: left ventricular end systolic volume; LV: left ventricle.*

Univariate regression analysis was performed to determine the variables which predict the LGE with CMR in HFrEF patients ( Table 4 ). Age, male sex, hyperlipidemia, coronary artery disease, QTc interval, Tp-e interval, QRS-T angle and plasma creatinine level were found to be significant. With these variables four different models were generated and multivariate regression analysis was done. In the first model all three QTc, Tp-e interval and QRS-T angle and other significant variables were included ( Table 4 ). In other three models, these parameters were evaluated separately ( Table 5 ). In first model male sex (p=0.032), coronary artery disease (p=0.009), plasma creatinine level (p=0037) and Tp-e interval [p=0.001, OR (95% CI) = 1.085 (1.032-1.140)] remained significant and they were independent predictors of LGE presence. In this model QTc interval (p=0.185) and QRS-T angle (p=0.944) lost their significance. In the model which evaluated only the QTc interval, QTc interval (p=0.007) was also found to be an independent predictor as well as male sex, coronary artery disease, plasma creatinine level. In the model which evaluated only the Tp-e interval, Tp-e interval remained significant (p<0.001) as in first model. In the model which evaluated only the QRS-T angle, male sex and coronary artery disease were found to be independent predictors but QRS-T angle couldn't reach the significance (p=0.058).

**Table 4:** – Univariate and multivariate regression analysis to determine the predictability of LGE in HF patients

Variable	Univariate analysis		Multivariate analysis	

	OR (95% CI)	p	OR (95% CI)	p
Age	1.051 (1.016-1.086)	0.004	0.960 (0.901-1.023)	0.209
Male	11.936 (3.230-44.114)	<0.001	6.348 (1.172-34.392)	0.032
Body mass index	0.927 (0.831-1.034)	0.173		
Diabetes mellitus	1.501 (0.615-3.668)	0.373		
Hypertension	0.958 (0.272-3.379)	0.947		
Hyperlipidemia	2.812 (1.224-6.464)	0.015	0.484 (0.105-2.226)	0.352
Coronary artery disease	8.036 (2.986-21.624)	<0.001	12.355 (1.851-82.445)	0.009
LBBB	0.841 (0.288-2.459)	0.752		
Atrial fibrillation	0.490 (1.110-2.176)	0.348		
QRS duration	0.996 (0.979-1.013)	0.646		
QTc interval	1.016 (1.006-1.027)	0.002	1.011 (0.995-1.028)	0.185
Tp-e interval	1.099 (1.055-1.144)	<0.001	1.085 (1.032-1.140)	0.001
QRS-T angle	1.017 (1.008-1.026)	<0.001	0.999 (0.984-1.015)	0.944
LVEF	0.976 (0.935-1.019)	0.273		
LVEDV index	1.003 (0.992-1.013)	0.637		
LVESV index	1.003 (0.991-1.013)	0.657		
Creatinine	18.678 (3.460-100.82)	0.001	12.501 (1.170-133.63)	0.037

*OR: odds ratio; CI: confidence interval; LBBB: left bundle branch block; LVEF: left ventricular ejection fraction; LVEDV: left ventricular end diastolic volume; LVESV: left ventricular end systolic volume.*

**Table 5:** – Multivariate regression analysis to determine the independent predictor of LGE in HF patients

Variable	Multivariate analysis					

	QTc interval		Tp-e interval		QRS-T angle	

	OR (95% CI)	p	OR (95% CI)	p	OR (95% CI)	p
Age	0.974 (0.921-1.030)	0.359	0.964 (0.905-1.027)	0.252	0.980 (0.927-1.036)	0.469
Male	6.393 (1.468-27.84)	0.013	7.405 (1.383-39.64)	0.019	7.369 (1.617-33.58)	0.010
Hyperlipidemia	0.643 (0.169-2.452)	0.518	0.513 (0.118-2.226)	0.626	0.727 (0.202-2.621)	0.398
Coronary artery disease	11.676 (2.43-56.09)	0.002	10.989 (1.89-63.69)	0.008	7.227 (1.605-32.55)	0.010
Creatinine	9.551 (1.548-58.92)	0.015	10.892 (1.26-94.01)	0.030	5.299 (0.947-29.66)	0.058
QTc, Tp-e, QRS-T angle	1.018 (1.005-1.032)	0.007	1.097 (1.045-1.152)	<0.001	1.010 (1.000-1.021)	0.058

*OR: odds ratio; CI: confidence interval.*

ROC curves for QTc, Tp-e interval and QRS-T angle were generated for LGE presence with CMR in HFrEF patients ( Figure 1 ). Although there is a need for confirmation with prospective studies, according to the ROC curves we obtained, the best QTc interval cut off value was 460.5 ms, Tp-e interval cut off value was 101.5 ms, and QRS-T angle cut off value was 110 degrees in the determination of HFrEF patients with LGE. When the patients were divided into two groups according to the Tp-e cut off which had the best AUC value, there were 54 patients (55.7%) in Tp-e≤101.5 group, 43 patients (44.3%) in Tp-e>101.5 group. In Tp-e>101.5 group, patients with LGE ratio were significantly higher than other group ( Figure 2 ). Similar to these findings, LGE segment numbers median values were significantly higher [5.0 (3.0-9.0) vs 0.0 (0.0-2.25), p<0.001] in Tp-e>101.5 group.

**Figure 1:** – Receiver operating characteristics curve showing the distinguishing ability of the QTc, Tp-e interval and QRS-T angle for LGE presence with CMR in HfrEF.

**Figure 2:** – Comparison of LGE rates in low and high Tp-e groups. HFrEF: Heart failure with reduced ejection fraction; LGE: late gadolinium enhancement.

## Discussion

In our study, we stated significantly higher QTc interval, Tp-e interval and QRS-T angle in HFrEF patients with LGE by CMR when compared to HFrEF patients without LGE. Also in correlation analysis, it was seen that the best correlation coefficients were found between CMR measurements which show cardiac structure and function and QRS-T angle; number of left ventricular segments with LGE and Tp-e intervals. In multivariate regression analysis which was performed by different models, Tp-e interval was found to be the best parameter within three parameters to predict the LGE presence in HFrEF patients. Also, Tp-e interval had the highest AUC value in ROC analysis performed for the LGE presence.

HF patients were divided into three groups according to the EF in latest European Society of Cardiology (ESC) guideline published in 2016. Group with EF under 40% was classified as HFrEF, group with EF 50% and more was classified as preserved EF HF (HFpEF), group with EF 40–49% was classified as mid-range EF HF (HFmrEF).^1^ The highest mortality rates are seen in HFrEF group.^21^ One of the important reasons of mortality is malignant arrhythmias. Myocardial fibrosis which is a substrate for these arrhythmias can be detected with LGE by CMR.^8^ In literature there are several studies showed the relationship between LGE presence and adverse cardiovascular events in HF patients.^9 - 11 , 22^

Liu et al. showed the relationship between LGE amount detected by CMR and major adverse cardiac events (MACE) in 84 stage C or D HF patients whether ischemic or not [p = 0.022, HR (95% CI) = 1.045 (1.001-1.084)].^11^ Shi et al.^22^ stated in their meta analysis which contained five studies and 545 dilated cardiomyopathy (DCM) patients, LGE presence was the predictor of high cardiovascular mortality [p=0.03, OR (95% CI) = 2.67 (1.12-6.35)], aborted SCD [p=0.007, OR (95% CI) = 5.26 (1.57-17.55)] and hospitalization due to HF [p<0.001, OR (95% CI) = 3.91 (1.99-7.69)].^22^ In Duan et al.^9^ meta analysis which evaluated 13 studies and included 1675 DCM patients, investigated the effects of LGE presence on MACE. In these 13 studies LGE presence ratios were between 18% and 71%. Results of analysis showed that LGE was associated with all cause mortality [p<0.001, OR (95% CI) = 3.43 (2.26-5.22)], cardiac death [p<0.001, OR (95% CI) = 3.65 (1.80-7.40)], hospitalization due to HF [p=0.001, OR (95% CI) = 2.87 (1.53-5.39)], major arrhythmic events [p<0.001, OR (95% CI) = 4.24 (2.95-6.08)] and SCD [p<0.001, OR (95% CI) = 3.33 (1.80-6.17)].^9^

Although CMR gives great informations to the clinicians, it has some limitations. CMR is not widely available, CMR interpretations need speciality, costs are high and safety for patients with metallic implants is still debated, can be less reliable in tachyarrhythmic patients, claustrophobia and contrast agent gadolinium usage in severe renal failure patients are other important limitations. So it can be a great convenience to predict myocardial fibrosis with repolarization parameters calculated from ECG which is easily available and interpretable. We built our study on this aim and we investigated whether QTc interval, Tp-e interval and frontal QRS-T angle are related with LGE presence and which one these parameters can be a better predictor.

QT and QTc intervals are the well known and largely used parameters of myocardial repolarization and are related to ventricular arrhythmia and cardiovascular mortality.^23^ In some studies it is shown that the duration between peak and end-point of T wave (Tp-e interval) is a new marker to evaluate the ventricular repolarization and because it isn't affected by heart rate variability, Tp-e interval can be more reliable to evaluate the ventricular repolarization. Moreover, Tp-e/QT and Tp-e/QTc ratio were shown to be a sensitive index of ventricular repolarization and arrhythmogenesis, as it provided an estimate of the dispersion of repolarization relative to the total duration of repolarization.^24 , 25^ Another new marker is spatial QRS-T angle. It is defined as angle difference between ventricular depolarization (QRS wave) direction and ventricular repolarization (T wave) direction.^18^ But calculation of the spatial QRS angle is too complicated and needs advanced computer programming.^26^ Conversely frontal QRS angle can be measured easily from automatic reports of ECG devices and has a good correlation with spatial QRS angle in risk stratification.^27^ That's reason why we used frontal QRS-T angle in our study.

Previous studies revealed in HF patients repolarization parameters were associated with all cause mortality, cardiac death, hospitalization due to HF, SCD and proper shocking in patients with ICD.^28 - 31^ Best of our knowledge there isn't any study investigating the relation of LGE with CMR and repolarization parameters so we aimed to meet the deficit. In our study, similar to the literature LGE ratio was 53.6% in HFrEF patients. All the repolarization parameters were significantly higher in patients with LGE. In univariate analysis all three parameters predicted LGE presence separately. In multivariate analysis firstly a model was generated which included all three repolarization parameters and the other significant variables. In this model only Tp-e interval from repolarization parameters remained significant and found to be an independent predictor of LGE presence [p=0.001, OR (95% CI) = 1.085 (1.032-1.140)]. With a different point of view, when we inserted the parameters separately to the models QTc interval and Tp-e interval remained significant but the significance level of Tp-e interval was better than the QTc interval (p<0.001 vs p=0.007). On the other hand Tp-e interval had medium correlation (r=0.564, p<0.001) with number of LV segments with LGE and other two parameters had poor correlations. As a result in our study Tp-e interval was found to be more related with the LGE in HFrEF patients when compared to other two repolarization parameters. Risk estimation of MACE in HFrEF patients can be possible with this parameter which can be easily measured from standard 12 lead ECG.

Our study had some limitations. First of all this study designed as a retrospective, single center and contained a small study population. No healthy volunteers were included to the study. There wasn't any clinical follow up data. HF patients were included to the study independent from the etiology. Evaluation of the patients separately according to the ischemic or non ischemic etiology could give more information. Our CMR data is limited in our study. LGE is only given as presence-absence and the number of segments showing involvement. The availability of additional data showing scar transmurality, percentage of LV scar and regional strain could enrich the study. Finally, in this study, cut-off values of repolarization parameters were developed using ROC curves. Therefore, our results must be interpreted carefully until they are confirmed in subsequent studies.

## Conclusion

In our study we found higher QTc interval, Tp-e interval and frontal QRS-T angle in HFrEF patients with LGE by CMR when compared to HFrEF patients without LGE. Tp-e interval was the best independent predictor of LGE presence. This information with the prolongation of the Tp-e interval allows us to predict the presence of myocardial fibrosis which is an arrhythmogenic substrate, in patients with HFrEF. As a result, we believe with a standard 12 lead ECG which is easily available and interpreted, it can be possible to predict the risk of adverse cardiovascular events in HFrEF patients and also we think decrease in MACE ratios can be obtained with intensive medical therapy, close follow-ups and ICD therapy. We know there should be more prospective, randomized, large populated studies to support our opinions.
